# A study on risk factors for the progression from T2DM to end-stage renal disease based on Mendelian randomization and logistic regression analysis

**DOI:** 10.1080/0886022X.2026.2687223

**Published:** 2026-08-05

**Authors:** Yakun Wang, Xinjia Guo, Yanhong Li, Qiyu Fu, Chong Zhang, Shoujun Bai

**Affiliations:** ^a^ Department of Nephrology, Qingpu Branch of Zhongshan Hospital Affiliated to Fudan University, Shanghai, China; ^b^ Department of Nephrology, Xin Hua Hospital Affiliated to Shanghai Jiao Tong University School of Medicine, Shanghai, China; ^c^ School of Digital Economics, Shanghai University of Finance and Economics, Shanghai, China; ^d^ Graduate School of Bengbu Medical University, Bengbu, Anhui Province, China

**Keywords:** Type 2 diabetes mellitus, end stage renal disease, Mendelian randomization, machine learning, risk factor

## Abstract

Using two-sample Mendelian randomization (MR) based on GWAS data from the IEU OpenGWAS project and a retrospective clinical cohort, this study investigated risk factors for progression from type 2 diabetes mellitus (T2DM) to end-stage renal disease (ESRD) and developed a predictive model. The T2DM GWAS dataset (ebi-a-GCST010118; 2020) included 433,540 individuals (77,418 cases and 356,122 controls). Multivariable MR, with inverse variance weighted as the primary method, was used to evaluate the causal effects of metabolic and hematologic traits on ESRD, while MR-Egger and Cochran’s Q test assessed pleiotropy and heterogeneity. MR-PRESSO was used for outlier removal. In parallel, 875 patients with T2DM were analyzed using univariable and multivariable logistic regression; 140 patients (16%) progressed to ESRD. MR showed that elevated body mass index (BMI) was a causal risk factor, whereas higher hematocrit was protective. In the clinical cohort, BMI, diastolic blood pressure, and creatinine were identified as independent risk factors, while albumin and hematocrit were protective. Sensitivity analyses showed no significant horizontal pleiotropy, and all instrumental variables were sufficiently strong (F-statistics >10). A logistic regression–based nomogram achieved an AUC of 0.88 (95% CI: 0.85–0.91) with good calibration and outperformed XGBoost, Random Forest, and support vector machine models. Elevated BMI and lower hematocrit increase ESRD risk in T2DM, and the nomogram may support early risk stratification and precision intervention.

## 1. Introduction

Diabetic nephropathy (DN) is one of the most common and severe chronic complications of diabetes and a leading cause of end‑stage renal disease (ESRD) and renal failure. As the global prevalence of diabetes continues to rise, the incidence and disease burden of DN have been increasing, making it a pressing public health concern [1,2]. The World Health Organization ranks diabetes as the fourth leading cause of death worldwide, and DN, as a major complication, significantly reduces patient survival rates and severely impairs quality of life [3]. This burden is particularly prominent in low‑ and middle‑income countries, largely due to the growing prevalence of diabetes, lifestyle changes, and population aging [4]. The International Diabetes Federation estimates that approximately 420 million people worldwide currently have diabetes, a number projected to rise to 700 million in the next two decades, with a corresponding increase in the risk of DN [5].

Epidemiological studies indicate that about 20%–40% of diabetic patients will develop DN during the course of the disease, particularly those with type 2 diabetes (T2DM), whose risk is closely associated with long‑term hyperglycemia exposure and insulin resistance [6,7]. Additionally, demographic factors such as race, gender, and age significantly influence the incidence of DN. Higher rates are observed among African Americans, Hispanics, and some Asian populations, often accompanied by hypertension, metabolic syndrome, and more rapid renal function decline [8,9].

Clinically, DN not only leads to proteinuria, renal tubular injury, and reduced renal function but also markedly increases the risk of complications such as cardiovascular disease, anemia, and electrolyte disturbances, making it the primary cause of ESRD [10]. Compared with the general population, DN patients have a significantly higher mortality rate, with approximately 60%–80% dying from cardiovascular events rather than renal failure itself [11]. Even with dialysis or kidney transplantation, patients still face high infection rates and severe complications, a significantly reduced quality of life, and a substantial burden on families and society [12].

As the adverse impacts of DN become increasingly evident, more research has focused on identifying its risk factors to provide a theoretical basis for early prevention and intervention. Substantial evidence indicates that physiological indicators, lifestyle factors, and medication use are closely associated with the onset and progression of DN [13–15]. For example, in patients with T2DM complicated by CKD, obesity exacerbates the progression of cardiovascular-kidney-metabolic syndrome, which integrates obesity, insulin resistance, CKD, and cardiovascular disease, significantly increasing morbidity and mortality[16–17]. However, establishing causal relationships between these factors remains methodologically challenging. Randomized controlled trials are considered the ‘gold standard’ for establishing causality but are often limited by high costs, feasibility issues, and ethical constraints, making them difficult to conduct in certain contexts [18–20].

Against this background, Mendelian randomization (MR) analysis has emerged as an important statistical method [21]. By using genetic variants as instrumental variables, MR can effectively reduce confounding and reverse causality often present in traditional observational studies [22], providing a robust framework for elucidating causal relationships between exposure factors and DN. The unique strength of MR lies in its use of genetics as a natural experiment, thereby enhancing the credibility of causal inference. For example, Gurung et al. using Mendelian randomization analysis, found that elevated plasma angiogenin levels are an independent risk factor for the development of ESKD in patients with type 2 diabetes; Park et al. found that excessive fat intake has a causal relationship with the progression of chronic kidney disease [23–24]. However, most existing studies lack further clinical validation for DN following MR analysis, limiting the interpretability and clinical applicability of causal findings in real‑world populations [25–26].

Therefore, in this study, we first employed MR analysis to clarify the causal relationships between specific exposures and the development of DN. Subsequently, to verify the robustness of these causal inferences, we designed and conducted a retrospective cohort study on the progression from T2DM to ESRD. Using real‑world clinical data, we applied multivariate logistic regression models to systematically analyze potential risk factors, thereby independently validating and supplementing the results obtained from MR analysis. By integrating genetic causal inference with clinical epidemiological evidence, this study not only strengthens the reliability of the conclusions but also provides a more solid scientific foundation for the prevention and intervention of diabetic nephropathy.

## 2. Materials and methods

### 2.1. Data sources for Mendelian randomization analysis

Genome-wide association study (GWAS) data for T2DM, ESRD, and various biochemical indicators were obtained from the IEU OpenGWAS project database (https://gwas.mrcieu.ac.uk/). Specifically, the T2DM data (accession ID: ebi-a-GCST010118) was published in 2020, with a sample size of 433,540 (comprising 77,418 cases and 356,122 controls) and 11,222,507 SNPs. Chronic renal failure (CRF) data was used as a proxy for ESRD. The CRF dataset (accession ID: ebi-a-GCST90018602) was published in 2021, with a sample size of 176,462 (comprising 2,117 cases and 174,345 controls) and 12,454,326 SNPs. All included SNPs were filtered using the ‘Population’ tag ‘East Asian’. A summary of the data is presented in Table 1.

**Table 1.** Summary of the GWAS studies.

Type	Trait/Variable	Year	ID	Sample Size	Number of SNPs
Exposure Factor I	T2DM	2020	ebi-a-GCST010118	433,540	11,222,507
Exposure Factor II	ALB	2021	ebi-a-GCST90018725	120,539	12,498,411
	Ca	2021	ebi-a-GCST90018731	83,980	12,492,704
	HCT	2021	ebi-a-GCST90018740	153,015	12,502,672
	SGLU	2021	ebi-a-GCST90018735	133,336	12,500,242
	CHO	2021	ebi-a-GCST90018754	135,808	12,500,351
	BMI	2021	ebi-a-GCST90018727	163,835	12,502,877
	UA	2021	ebi-a-GCST90018757	129,405	12,499,459
	HBA1C	2020	ukb-e-30750_EAS	2,566	8,251,701
	DBP	2021	ebi-a-GCST90018732	145,515	12,501,486
	SBP	2021	ebi-a-GCST90018752	145,505	12,501,486
	CRP	2021	ebi-a-GCST90018730	83,025	12,493,987
	WBC	2021	ebi-a-GCST90018758	154,355	12,502,795
	LDH	2019	bbj-a-30	126,319	6,108,953
Outcome	CRF	2021	ebi-a-GCST90018602	176,462	12,454,326

Abbreviations: T2DM, Type 2 diabetes mellitus; ALB, Albumin; Ca, Calcium; HCT, Hematocrit; SGLU, Serum glucose; CHO, Cholesterol; BMI, Body mass index; UA, Uric acid; HBA1C, Glycated hemoglobin; DBP, Diastolic blood pressure; SBP, Systolic blood pressure; CRP, C-reactive protein; WBC, White blood cell count; LDH, Lactate dehydrogenase; CRF, Chronic renal failure.

### 2.2. Instrumental variable (IV) selection

The screening threshold for single nucleotide polymorphisms (SNPs) was set at *p* < 5 × 10^−8^. To ensure the independence of the selected SNPs and avoid pleiotropy, the clumping parameters were set with a linkage disequilibrium (LD) coefficient R^2^ < 0.001 and a physical distance window of 10,000 kb. Subsequently, the formula $F=\frac{N-K-1}{K}\times \frac{R^{2}}{1-R^{2}}$>10 was applied to exclude weak instrumental variables, ensuring a strong association between the IVs and the exposure factors. In this formula, N represents the sample size, K is the number of SNPs (*K* = 1 for individual SNP validation), and R^2^ is the proportion of variance explained by the SNP in the GWAS summary statistics. Here, the number of instruments (K) was set to 1 (*K* = 1) at the individual SNP level to assess the strength of each genetic variant separately. The overall (set-level) F-statistic for all instruments combined (e.g. for BMI, *F* = 19.70), where K equals the total number of SNPs in the instrumental set, was reported in the supplementary forest plot to evaluate the global strength of the instrumental variables. The LDlink and FastTraitR tools were employed to remove potential confounding effects associated with the genetic instrument variable loci.

### 2.3. Mendelian randomization (MR) analysis

Statistical analysis was performed using the TwoSampleMR package (version 0.7.0) in R. The primary method for assessing the causal effect of multiple SNPs on the outcome was the Inverse Variance Weighted (IVW) method, which works by performing a meta-analysis of the Wald ratios for each individual SNP. Under the assumption of no significant horizontal pleiotropy, the IVW method provides a more robust and precise estimate of the causal effect compared to other methods and was therefore used as the primary analytical approach in this study. Results were interpreted using Odds Ratios (OR) and their 95% Confidence Intervals (95% CI). An OR > 1 indicates that the exposure is a risk factor, while 0 < OR < 1 suggests a protective factor. To test for the presence of horizontal pleiotropy, MR-Egger regression was employed. This method includes an intercept term in its model, and a *P*-value > 0.05 for the intercept indicates no significant horizontal pleiotropy. MR-PRESSO was additionally used to identify specific outlier single nucleotide polymorphisms (SNPs). In this method, if the global test P-value was < 0.05, indicating significant pleiotropy, the outlier-corrected causal estimate was reported after removing the detected outliers. This approach provides a more robust estimate in the presence of horizontal pleiotropy. Heterogeneity among the instrumental variable estimates was assessed using Cochran’s Q test. A P-value < 0.05 suggested the presence of heterogeneity, in which case the random-effects IVW model was applied; otherwise, the fixed-effects IVW model was used. Furthermore, funnel plots were generated to examine the reliability of the causal estimates, and forest plots were created to visually present the causal associations between various factors and end-stage renal disease.

Leave-one-out analysis was conducted to identify influential SNPs. Each SNP was sequentially removed, and the causal effect was re-estimated using IVW on the remaining SNPs. A substantial change in the estimate or a confidence interval crossing the null (OR = 1) would indicate disproportionate influence by that SNP. Stable estimates across iterations support robustness. Results were visualized using a leave-one-out plot.

In univariable MR analysis, genetic variants associated with T2DM may influence ESRD risk indirectly through their effects on adiposity-related traits (such as BMI and blood pressure), potentially leading to biased estimates. To address the potential confounding effect of T2DM, we employed multivariable Mendelian randomization (MVMR) by including both T2DM and each adiposity trait as exposures in the model. This approach was not intended to replicate the causal effect of T2DM on ESRD, but rather to control for T2DM as a strong confounder at the genetic level, thereby enabling a more accurate estimation of the independent causal effects of each adiposity trait on ESRD.

Theoretically, conducting a subgroup analysis restricted to individuals with T2DM would be ideal. However, due to the current lack of large-scale genome-wide association study (GWAS) summary data specifically for the T2DM population, a direct subgroup MR analysis was not feasible.

Decision curve analysis was performed to evaluate the clinical utility of the ESRD prediction model. Net benefit was calculated across threshold probabilities ranging from 0% to 50%.

To assess the internal validity and stability of the prediction model, bootstrap resampling with 500 iterations was performed. The optimism-corrected Area Under the Receiver Operating Characteristic Curve (AUC) was calculated, along with 95% confidence intervals (CI) using the percentile method.

### 2.4. Data source for the retrospective cohort study

To validate the results obtained from the Mendelian Randomization analysis, a retrospective cohort study was conducted. Based on predefined inclusion and exclusion criteria, a total of 875 patients diagnosed with T2DM at Qingpu Branch of Zhongshan Hospital, Fudan University between January 1, 2016, and December 31, 2023, were enrolled as study subjects. All patients were followed up for 3 years. The study endpoint was progression to ESRD. The cohort comprised 140 patients who progressed to ESRD and 735 patients who did not.

Inclusion Criteria: (1) Age ≥ 18 years; (2) Diagnosis of T2DM meeting the WHO (1999) diagnostic and classification criteria as outlined in the ‘Chinese Guidelines for the Prevention and Treatment of Type 2 Diabetes (2013 Edition)’.

Exclusion Criteria: (1) Specific types of diabetes (e.g. Type 1 diabetes, secondary diabetes). (2) Presence of acute diabetic complications such as diabetic ketoacidosis or hyperosmolar hyperglycemic state. (3) Renal biopsy findings indicating primary glomerular diseases, or systemic diseases potentially causing secondary renal damage (e.g. systemic lupus erythematosus, vasculitis, Henoch-Schönlein purpura). (4) Patients who had already initiated peritoneal dialysis, hemodialysis, or received a kidney transplant. (5) Patients with concurrent urinary tract or systemic infections, cardiac or hepatic insufficiency, or major post-operative status. (6) Patients with concurrent malignancies, pregnancy, or severe psychiatric disorders. (7) Patients with a solitary kidney. (8) Patients with kidney damage due to drugs, heavy metals, or other nephrotoxic substances, or those on long-term immunosuppressive therapy. (9) Patients with incomplete clinical data or loss to follow-up.

### 2.5. Ethics approval and patient consent

All studies involving human participants and data were conducted in accordance with the principles set forth in the Declaration of Helsinki. The retrospective clinical cohort study was reviewed and approved by the Ethics Committee of Qingpu Branch of Zhongshan Hospital Affiliated to Fudan University (Approval Number: QY 2021-43). Written informed consent was obtained from all participants or their legal guardians. This written consent was chosen to ensure a clear, documented agreement from participants regarding the use of their data and their participation in the study, adhering to standard ethical research practices. For the Mendelian randomization analysis, ethical approval and patient consent were obtained in the original genome-wide association studies (GWAS), and the data used are publicly available and de-identified.

### 2.6. Observation indicators and methods

This study collected patients’ general information and laboratory indicators. General information included baseline characteristics such as age, gender, systolic blood pressure (SBP), diastolic blood pressure (DBP), height, weight, and calculated body mass index (BMI). Laboratory indicators included C-reactive protein (CRP), albumin (Alb), total cholesterol (CHO), serum creatinine (Scr), calcium (Ca), glycated hemoglobin (HbA1c), hematocrit (HCT), lactic dehydrogenase (LDH), serum glucose (SGLU), and uric acid (UA).

### 2.7. Statistical methods

Data organization was performed using the pandas (version 2.0.3) and sklearn (version 1.2.2) packages in Python. Baseline characteristic tables were generated using the tableone package (version 0.13.2) in R. For missing values in the clinical data, if the missing rate for a continuous variable was less than 5%, multiple imputation was performed to impute missing values; if the missing rate was higher than 5%, the variable was excluded from the primary analysis and used only for descriptive analysis. Before constructing the prediction models (including logistic regression, XGBoost, Random Forest, and Support Vector Machine), all continuous variables were standardized to eliminate the influence of dimensions and ensure model convergence and comparability. Logistic regression analysis was conducted using the stats package in R. Normally distributed continuous data are presented as mean ± standard deviation and compared between groups using the Student’s t-test. Non-normally distributed continuous data are presented as median (Q25, Q75) and compared using the Mann-Whitney U test. Categorical data are presented as counts and percentages and compared using the Chi-square test. Variables with a *P*-value < 0.05 in the univariate logistic regression analysis were included in the multivariate logistic regression model. The Variance Inflation Factor (VIF) was calculated to test for potential multicollinearity among the risk factors. The discriminatory performance of the model was evaluated using the Receiver Operating Characteristic (ROC) curve and the Area Under the Curve (AUC). A nomogram was constructed based on the logistic regression results, and its accuracy was validated using a standard calibration curve. Furthermore, the dataset was randomly split into a training set (70%) and a testing set (30%). Prediction models were built using eXtreme Gradient Boosting (XGBoost), Random Forest (RF), and Support Vector Machine (SVM) algorithms. Sensitivity and specificity were calculated, and the performance of these models was comprehensively compared. In the Hosmer Lemeshow test, a P-value greater than 0.05 usually indicates a good fit of the model.

## 3. Results

### 3.1. MR analysis

The study included 14 instrumental variables, including T2DM. The F-statistics for all instrumental variables were greater than 10, indicating the absence of weak instrument bias. A two-sample Mendelian randomization analysis was conducted using T2DM and other demographic variables and biochemical indicators as exposures, and chronic renal failure as the outcome variable. Heterogeneity among the instrumental variables was assessed using Cochran’s Q test. When the P-value was > 0.05, it indicated no significant heterogeneity, and the fixed-effects IVW model was used for causal inference. If the *P*-value was ≤ 0.05, the random-effects IVW model was applied. In this study, BMI showed some heterogeneity, and thus the random-effects model was used for their analysis. As shown in Table 2, T2DM, hematocrit, glucose, and BMI showed significant causal associations with chronic renal failure (*p* < 0.05), with 95% confidence intervals that did not cross 1. Furthermore, the pleiotropy test results indicated no significant horizontal pleiotropy (*P* for Egger intercept > 0.05).

**Table 2.** MR analysis results for each indicator and renal failure.

Phenotype	Method	Renal Failure			
		SNP	B	SE	P
Type 2 Diabetes Mellitus	Inverse variance weighted	149	0.2559636	0.03987413	1.369130e-10
	MR Egger	149	0.3074244	0.08677995	5.303874e-04
	Weighted median	149	0.2194899	0.06704856	0.001061882
	Simple mode	149	0.1451897	0.16842511	3.900583e-01
	Weighted mode	149	0.2824918	0.0944993	3.271369e-03
Serum Glucose	Inverse variance weighted	18	0.8778583	0.2500292	0.000446386
	MR Egger	18	0.4223361	1.1708177	0.723027813
	Weighted median	18	0.7629194	0.3535246	0.030924908
	Simple mode	18	0.7818822	0.6022274	0.211516232
	Weighted mode	18	0.7569734	0.5733985	0.204284534
Diastolic Blood Pressure	Inverse variance weighted	24	0.24184877	0.2997899	0.41982351
	MR Egger	24	−0.09468124	1.1408911	0.93461088
	Weighted median	24	0.93513866	0.393001	0.01733702
	Simple mode	24	1.31506062	0.7523808	0.09382641
	Weighted mode	24	1.36172934	0.823423	0.11176552
Body Mass Index	Inverse variance weighted	77	0.4888514	0.2037906	0.016449233
	MR Egger	77	0.9455067	0.58602	0.110849506
	Weighted median	77	0.8611816	0.2430751	0.000395811
	Simple mode	77	1.5092064	0.6964721	0.033373509
	Weighted mode	77	1.352593	0.3650566	0.000398687
Uric Acid	Inverse variance weighted	58	0.07880123	0.144387	0.585228074
	MR Egger	58	−0.36390583	0.2405933	0.13602121
	Weighted median	58	−0.36225562	0.1491838	0.01517187
	Simple mode	58	−0.21284579	0.3049587	0.48804626
	Weighted mode	58	−0.36454054	0.1400313	0.01175059
Glycated Hemoglobin A1c	Inverse variance weighted	0			
	MR Egger	0			
	Weighted median	0			
	Simple mode	0			
	Weighted mode	0			
Hematocrit	Inverse variance weighted	52	−0.6678943	0.2377855	0.004972504
	MR Egger	52	−0.6671163	0.6183588	0.285831826
	Weighted median	52	−0.6978385	0.3211126	0.029766046
	Simple mode	52	−0.3118575	0.7350542	0.673157183
	Weighted mode	52	−0.8802052	0.477159	0.070895428
C-Reactive Protein	Inverse variance weighted	9	0.2754662	0.2011425	0.1708406
	MR Egger	9	0.4285367	0.4010498	0.3207356
	Weighted median	9	0.2831334	0.2511159	0.2595309
	Simple mode	9	0.2080137	0.3697108	0.5890956
	Weighted mode	9	0.3706888	0.2708509	0.2083172
Systolic Blood Pressure	Inverse variance weighted	38	0.3104297	0.2485014	0.21158944
	MR Egger	38	0.4879536	0.9769342	0.62048423
	Weighted median	38	0.7013454	0.3244529	0.0306471
	Simple mode	38	1.2131222	0.7602211	0.11905206
	Weighted mode	38	1.2131222	0.7158845	0.09855782
Lactate Dehydrogenase	Inverse variance weighted	15	0.208224	0.1051099	0.04758976
	MR Egger	15	0.2183883	0.1437192	0.15256613
	Weighted median	15	0.2949257	0.1320337	0.02550179
	Simple mode	15	0.1070335	0.3091481	0.73432496
	Weighted mode	15	0.2685991	0.1184371	0.03969272
Calcium	Inverse variance weighted	15	−0.123277	0.2336371	0.597747
	MR Egger	15	0.50339498	1.0954133	0.6534312
	Weighted median	15	−0.03988532	0.3241413	0.9020682
	Simple mode	15	−0.45165056	0.6034954	0.4666118
	Weighted mode	15	−0.0466898	0.4904934	0.9255134
Cholesterol	Inverse variance weighted	53	0.006389091	0.1414226	0.963966
	MR Egger	53	−0.01139497	0.2764636	0.9672839
	Weighted median	53	−0.123985269	0.190778	0.5157614
	Simple mode	53	−0.009968727	0.3075143	0.9742635
	Weighted mode	53	−0.12900147	0.2064931	0.5348831
Creatinine	Inverse variance weighted	84	0.5952722	0.17975	0.000927419
	MR Egger	84	0.3812192	0.5338819	0.477222994
	Weighted median	84	0.5327823	0.2463414	0.030558083
	Simple mode	84	0.3901083	0.6582007	0.555000708
	Weighted mode	84	0.3345955	0.5645929	0.555038989
Albumin	Inverse variance weighted	30	−0.08513383	0.2650198	0.7480317
	MR Egger	30	−0.05090644	0.7469663	0.9461499
	Weighted median	30	−0.06303548	0.2942172	0.8303535
	Simple mode	30	−0.31683364	0.5255556	0.5512912
	Weighted mode	30	−0.22839225	0.3724316	0.5444937

**Figure 1.**
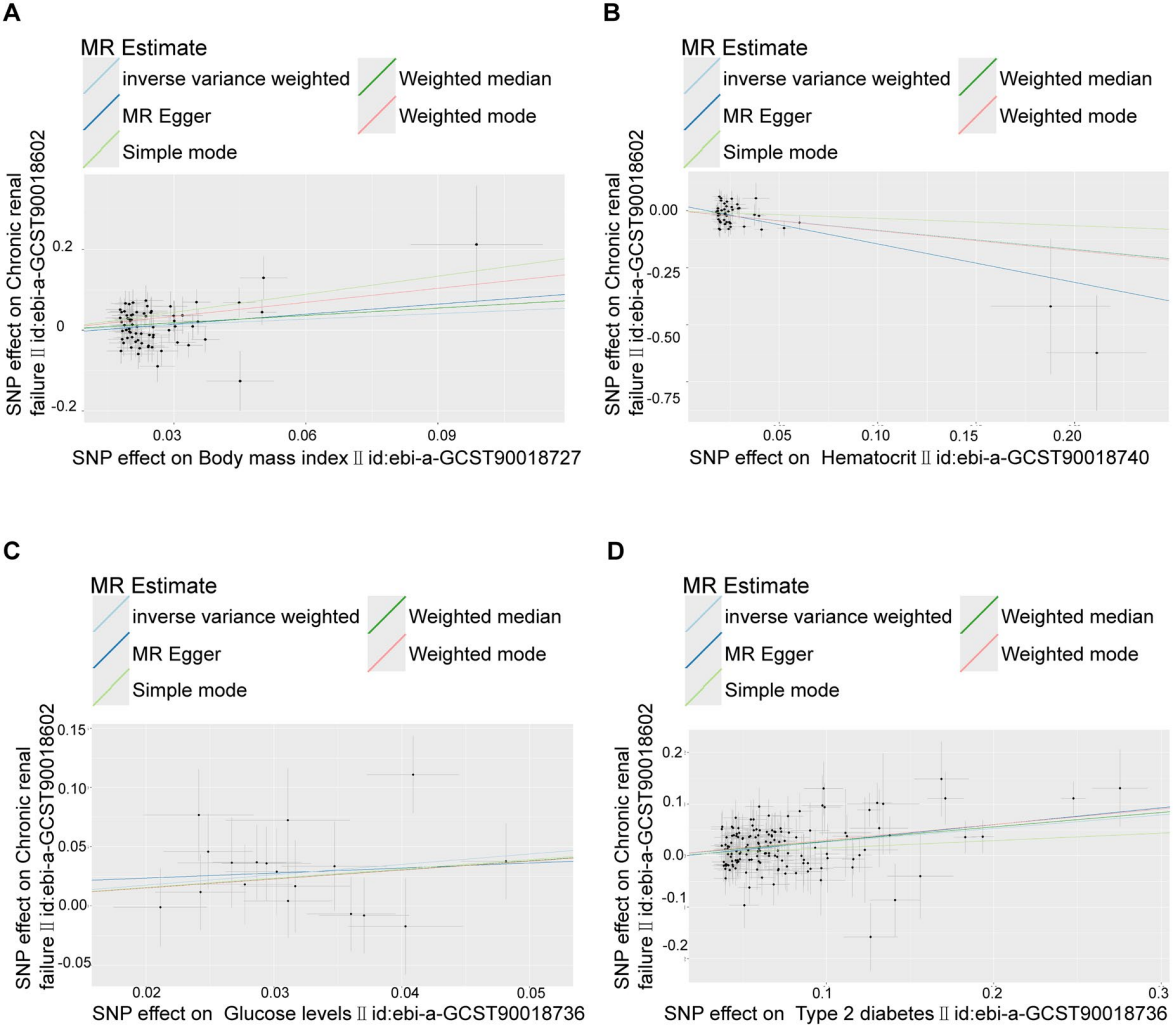
Mendelian randomization analysis of different metabolism-related indicators on the risk of chronic renal failure. (A) Causal effect of body mass index (BMI) on chronic renal failure risk; (B) Causal effect of hematocrit on chronic renal failure risk; (C) Causal effect of glucose levels on chronic renal failure risk; (D) Causal effect of T2DM on chronic renal failure risk.

Further analysis revealed that a genetically predicted 1-standard-deviation (SD) increase in glucose level was associated with higher odds of chronic renal failure (OR = 2.406, 95% CI = 1.470–3.930, *p* = 0.00044). Similarly, a genetically predicted 1-SD increase in BMI was associated with higher odds of chronic renal failure (OR = 1.630, 95% CI = 1.090–2.430, *p* = 0.016), while greater genetic liability to T2DM was also associated with higher odds of chronic renal failure (OR = 1.291, 95% CI = 1.190–1.400, *p* = 1.37 × 10⁻¹⁰). In contrast, a genetically predicted 1-SD increase in hematocrit was associated with lower odds of chronic renal failure (OR = 0.513, 95% CI = 0.320–0.820, *p* = 0.00497; Figure 1).

To further detect and correct for potential outliers, the MR-PRESSO test was performed. For hematocrit, three outlier SNPs (rs10168349, rs855791, rs1658435) were identified and removed, after which the global test showed no significant pleiotropy (*p* > 0.05). For BMI, eight outlier SNPs (rs2237897, rs764129, rs11916906, rs3814424, rs6567160, rs11642015, rs1996023, rs13019219) were detected and removed, yielding a non-significant global test (*p* > 0.05). Following outlier removal, the causal estimates remained consistent with the primary analysis.(see Table 3).

**Table 3.** MR-PRESSO test.

Exposure	Outcome	Inverse variance weighted						MR-PRESSO Test P-Value	IVW heterogeneity test	
		SNP	β	SE	P	OR	95%CI		Q	P
Glucose (per 1-SD increase)	chronic renal failure	18	0.8778583	0.2500292	0.000446386	2.406	1.470–3.930	0.561	15.28210	0.5751705
Body Mass Index (per 1-SD increase)	chronic renal failure	69	0.4568324	0.1887754	0.01552143	1.579064	1.091-2.286	0.052	87.76786	0.05369953
Genetic liability to Type-2 Diabetes Mellitus	chronic renal failure	149	0.2559636	0.03987413	1.369130*10^10^	1.291	1.190–1.400	0.543	146.0331	0.5303024
Hematocrit (per 1-SD increase)	chronic renal failure	49	−0.8376437	0.2163157	0.0001078044	0.4327290	0.283-0.661	0.109	61.94738	0.08507574

**Figure 2.**
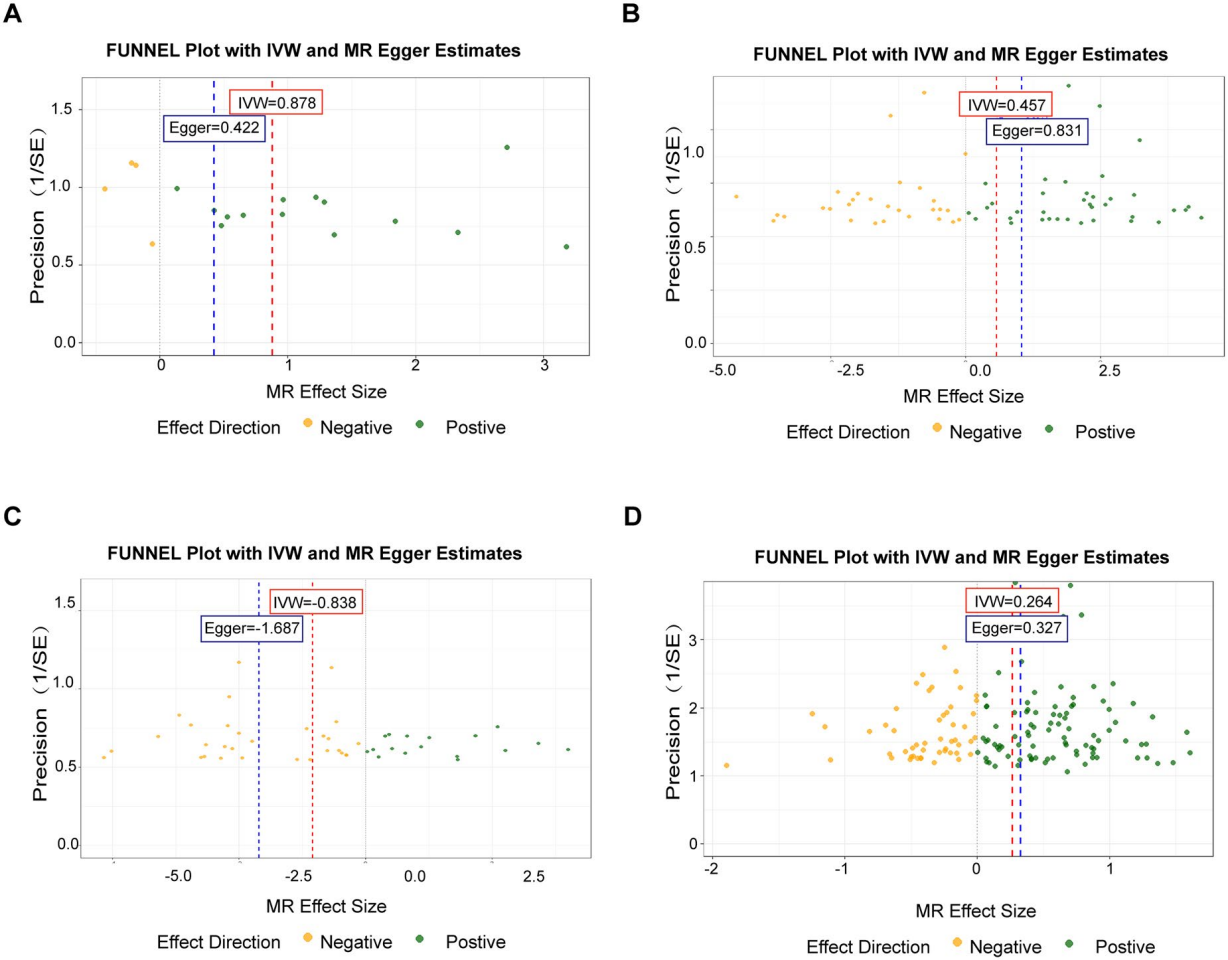
Funnel plots of MR analysis for different metabolic and disease factors on chronic renal failure. (A) MR funnel plot for glucose levels and chronic renal failure; (B) MR funnel plot for BMI and chronic renal failure; (C) MR funnel plot for hematocrit and chronic renal failure; (D) MR funnel plot for T2DM and chronic renal failure.

In the funnel plots of the MR analysis (Figure 2A–D), the data points were roughly symmetrically distributed on both sides of the causal effect estimate line, collectively forming a symmetrical pyramid shape. The effect estimates from the IVW and MR-Egger methods were highly consistent, indicating that the results of this study were not significantly affected by horizontal pleiotropy or heterogeneity. The causal effect estimates are relatively robust, and the conclusions are reliable.

**Figure 3.**
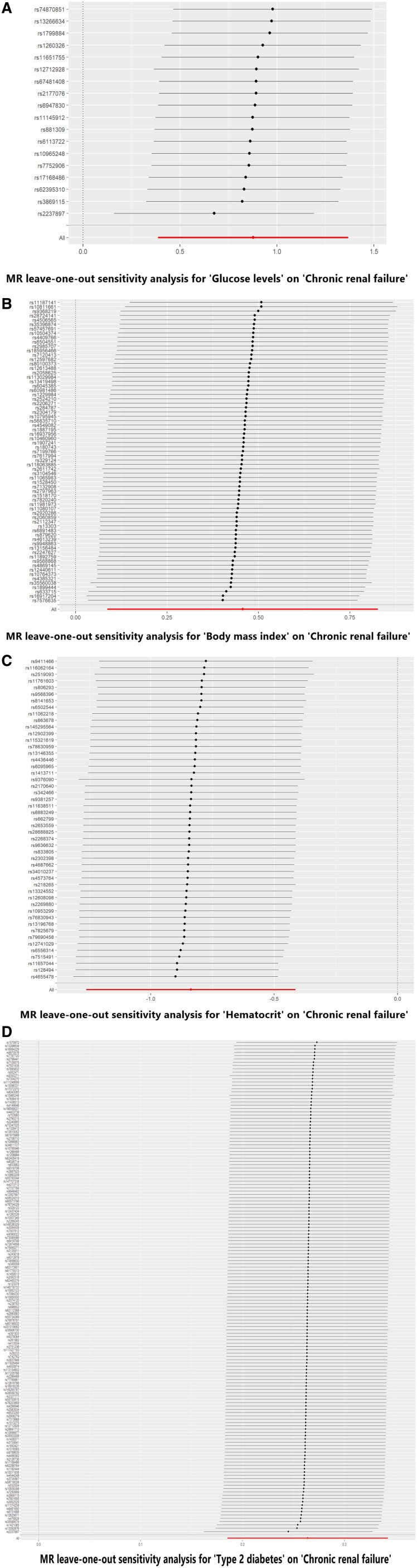
Leave-one-out test of causal associations between different metabolic and disease factors on chronic renal failure. (A) MR leave-one-out sensitivity analysis for Glucose levels; (B) MR leave-one-out sensitivity analysis for BMI; (C) MR leave-one-out sensitivity analysis for hematocrit; (D) MR leave-one-out sensitivity analysis for T2DM.

A leave-one-out sensitivity analysis was performed, as shown in Figure 3. The consistency of the causal effect sizes across all leave-one-out scenarios demonstrates the robustness of the causal inference between different metabolic and disease factors on chronic renal failure.

### 3.2. Multivariable MR analysis

**Figure 4.**
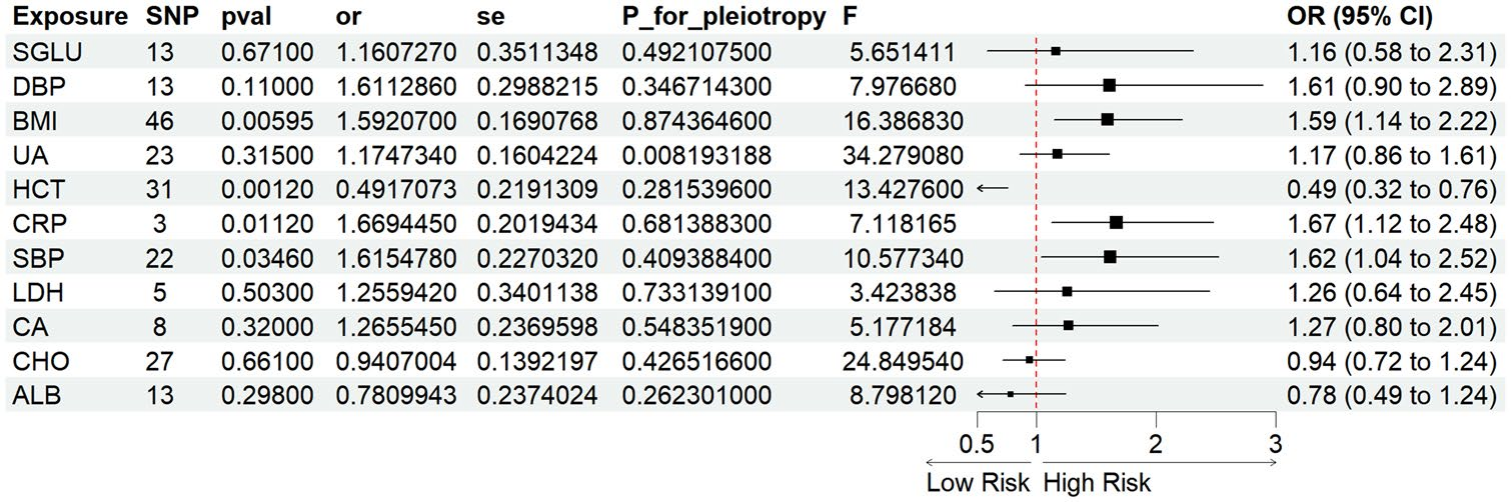
Multivariable Mendelian randomization analysis. Abbreviations: ALB, albumin; BMI, body mass index; CA, ­calcium; CHO, total cholesterol; CRP, C-reactive protein; DBP, diastolic blood pressure; HCT, hematocrit; LDH, lactate dehydrogenase; SBP, systolic blood pressure; SGLU, serum glucose; UA, uric acid.

To further explore the relationships between T2DM-related indicators and end-stage renal disease, this study incorporated the SNPs for the aforementioned indicators along with those for T2DM into a multivariable Mendelian randomization analysis. No eligible SNPs for HbA1c satisfied the genome-wide significance threshold (*p* < 5 × 10^−8^); therefore, HbA1c was not included in Figure 4. After adjustment for genetic liability to T2DM, each genetically predicted 1-SD increase in BMI was associated with 59.2% higher odds of chronic renal failure (OR = 1.592, 95% CI = 1.143–2.218, p = 5.95 × 10⁻³), whereas each genetically predicted 1-SD increase in hematocrit was associated with 50.8% lower odds of chronic renal failure (OR = 0.492, 95% CI = 0.320–0.756, p = 1.20 × 10⁻³) remained a protective factor. The F-statistics for all instrumental variables were greater than 10, indicating the absence of weak instrument bias in this analysis.

SGLU was excluded from the multivariable Mendelian randomization analysis because its corresponding F-statistic (5.65) fell below the conventional threshold of 10, indicating a weak instrument bias. Additionally, the P-value for its association with the outcome was far above the significance level (*p* = 0.671), suggesting no meaningful effect.

### 3.3. General baseline characteristics of the retrospective cohort study

A total of 875 patients were included in the study, of whom 735 did not progress to ESRD and 140 progressed to ESRD. Compared with the non-progressor group, the ESRD progressor group had higher levels of alkaline phosphatase (ALP), BMI, CREA, DBP, SBP, CRP, UA, and SGLU. Conversely, CHO, ALB, HCT, CA, HbA1c, and LDH levels were lower in the progressor group, as detailed in Table 4.

**Table 4.** Baseline characteristics.

Variables	Total (*n* = 875)	Non-progressor group (*n* = 735)	ESRD progressor group (*n* = 140)	Statistic	*P*
Body mass index (kg/m²), Mean ± SD	25.335 ± 3.809	25.215 ± 3.786	25.964 ± 3.881	t=-2.135	0.033
Systolic blood pressure (mmHg), Mean ± SD	138.570 ± 20.574	136.725 ± 20.105	148.259 ± 20.363	t=-6.209	<.001
Diastolic blood pressure (mmHg), Mean ± SD	75.959 ± 12.691	75.721 ± 12.863	77.204 ± 11.708	t=-1.268	0.205
Albumin (g/L), Mean ± SD	37.506 ± 5.339	38.115 ± 5.095	34.306 ± 5.463	*t* = 8.012	<.001
Alkaline phosphatase (U/L), Mean ± SD	84.450 ± 34.505	83.720 ± 35.370	88.283 ± 29.369	t=-1.435	0.152
Calcium (mmol/L), Mean ± SD	2.223 ± 0.143	2.233 ± 0.138	2.173 ± 0.156	*t* = 4.592	<.001
Total cholesterol (mmol/L), Mean ± SD	4.595 ± 1.571	4.598 ± 1.675	4.581 ± 0.840	*t* = 0.184	0.854
Serum creatinine (μmol/L, Mean ± SD	93.597 ± 62.235	80.504 ± 42.003	162.337 ± 96.811	t=-9.827	<.001
C-reactive protein (mg/L), Mean ± SD	11.671 ± 20.256	11.602 ± 20.896	12.036 ± 16.550	t=-0.232	0.817
Glycated hemoglobin (%), Mean ± SD	8.350 ± 2.030	8.378 ± 2.010	8.204 ± 2.133	*t* = 0.932	0.352
Hematocrit (%), Mean ± SD	38.781 ± 4.886	39.479 ± 4.549	35.116 ± 4.971	*t* = 10.244	<.001
Lactate dehydrogenase (U/L), Mean ± SD	297.146 ± 519.099	307.747 ± 560.083	241.491 ± 184.747	*t* = 1.385	0.166
Serum glucose (mmol/L), Mean ± SD	9.248 ± 4.346	9.222 ± 4.314	9.381 ± 4.524	t=-0.397	0.692
Uric acid (μmol/L), Mean ± SD	354.851 ± 114.283	345.792 ± 112.137	402.407 ± 114.055	t=-5.460	<.001
Age (years), M(Q_25_, Q_75_)	68 (60, 75)	68 (60, 75)	66 (59, 72)	Z=-1.71	0.087
Sex, n (%)				χ²=1.39	0.239
Female	358 (40.91)	307 (41.77)	51 (36.43)		
Male	517 (59.09)	428 (58.23)	89 (63.57)		

Note: SD, standard deviation; Q25, 25th percentile; Q75, 75th percentile; and ESRD, end-stage renal disease.

### 3.4. Analysis of influencing factors

As shown in Table 5, univariable logistic regression analysis indicated that BMI (*p* = 0.034), systolic blood pressure (*p* < 0.001), albumin (*p* < 0.001), calcium (*p* < 0.001), serum creatinine (*p* < 0.001), hematocrit (*p* < 0.001), and uric acid (p < 0.001) were significantly associated with progression to end-stage renal disease (ESRD). After assessment of multicollinearity (all VIFs < 10), the associations of uric acid and calcium were no longer significant in the multivariable logistic regression analysis. Each 1-kg/m² increase in BMI was associated with 8.5% higher odds of ESRD progression (OR = 1.085, 95% CI = 1.026–1.147, *p* = 0.004), each 1-mmHg increase in systolic blood pressure was associated with 2.0% higher odds (OR = 1.020, 95% CI = 1.008–1.032, *p* < 0.001), and each 1-μmol/L increase in serum creatinine was associated with 1.8% higher odds (OR = 1.018, 95% CI = 1.014–1.022, *p* < 0.001). Conversely, each 1-percentage-point increase in hematocrit was associated with 6.0% lower odds of ESRD progression (OR = 0.940, 95% CI = 0.891–0.993, *p* = 0.027), and each 1-g/L increase in albumin was associated with 4.7% lower odds (OR = 0.953, 95% CI = 0.913–0.995, *p* = 0.027). The association between higher hematocrit and improved kidney outcomes may be explained by several potential mechanisms. SGLT2 inhibitors reduce the metabolic load and oxidative stress in proximal tubular cells by inhibiting tubular glucose reabsorption, thereby stimulating renal erythropoietin (EPO) production, promoting erythropoiesis, and moderately increasing hematocrit. This response may reflect enhanced erythropoiesis rather than hemoconcentration alone. A moderate increase in hematocrit may also improve renal medullary oxygen delivery. In addition, computational fluid dynamics studies suggest that, within the physiological range, hematocrit and red blood cell aggregation can influence blood viscosity under pulsatile flow and may thereby affect microcirculatory perfusion.

**Table 5.** Logistic regression results.

Variables	Univariable analysis					Multivariate analysis				
	β	S.E	Z	*P*	OR (95%CI)	β	S.E	Z	*P*	OR (95%CI)
Body mass index (kg/m²)	0.049	0.023	2.119	0.034	1.050 (1.004–1.098)	0.082	0.028	2.879	0.004	1.085 (1.026–1.147)
Systolic blood pressure (mmHg)	0.030	0.005	6.110	<.001	1.031 (1.021–1.041)	0.020	0.006	3.362	<.001	1.020 (1.008–1.032)
Diastolic blood pressure (mmHg)	0.010	0.007	1.276	0.202	1.010 (0.995–1.024)					
Albumin (g/L)	−0.122	0.017	−7.199	<.001	0.885 (0.857–0.915)	−0.048	0.022	−2.205	0.027	0.953 (0.913–0.995)
Calcium (mmol/L)	−2.997	0.665	−4.505	<.001	0.050 (0.014– 0.184)					
Total cholesterol (mmol/L)	−0.007	0.061	−0.119	0.905	0.993 (0.881–1.118)					
Serum creatinine (μmol/L)	0.021	0.002	10.436	<.001	1.021 (1.017– 1.025)	0.018	0.002	8.232	<.001	1.018 (1.014–1.022)
C-reactive protein (mg/L)	0.001	0.004	0.232	0.816	1.001 (0.992–1.010)					
Glycated hemoglobin (%)	−0.044	0.047	−0.932	0.351	0.957 (0.872–1.050)					
Hematocrit (%)	−0.186	0.021	−8.892	<.001	0.830 (0.797–0.865)	−0.062	0.028	−2.217	0.027	0.940 (0.891– 0.993)
Lactate dehydrogenase (U/L)	−0.001	0.000	−1.911	0.056	0.999 (0.998–1.000)					
Serum glucose (mmol/L)	0.008	0.021	0.397	0.691	1.008 (0.968–1.050)					
Uric acid (μmol/L)	0.004	0.001	5.130	<.001	1.004 (1.002–1.005)					

Note: β, regression coefficient; SE, standard error; Z, Wald Z statistic; OR, odds ratio; CI, confidence interval. The ORs are expressed per one-unit increase in each variable according to the measurement units shown in the Variables column.

### 3.5. Model evaluation

**Figure 5.**
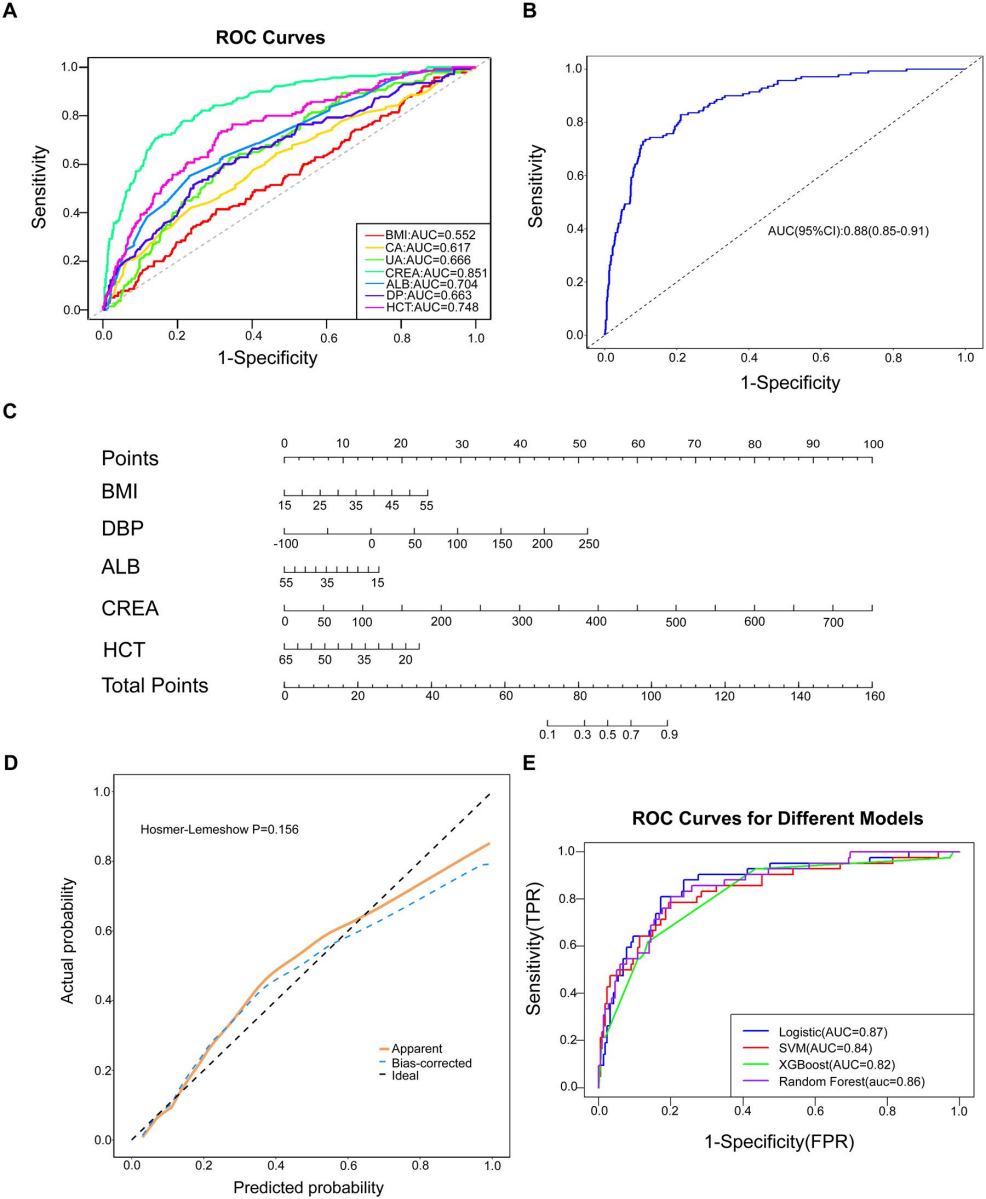
Evaluation of the end-stage renal disease risk prediction model based on logistic regression. (A) Receiver operating characteristic (ROC) curves for individual predictors; (B) ROC curve for the multivariable logistic regression model; (C) nomogram based on the multivariable logistic regression model; (D) calibration curve and Hosmer–Lemeshow goodness-of-fit test; and (E) ROC curves comparing the different prediction models. Example of nomogram use: for a patient with a BMI of 28 kg/m², DBP of 90 mmHg, ALB of 35 g/L, CREA of 100 μmol/L, and HCT of 40%, the total score is 175, corresponding to an estimated risk of ESRD progression of approximately 65%. Abbreviations: ALB, albumin; AUC, area under the curve; BMI, body mass index; CREA, serum creatinine; ESRD, end-stage renal disease; HCT, hematocrit; ROC, receiver operating characteristic; DBP, diastolic blood pressure.

The results showed that the ROC curves for different individual variables exhibited differential predictive performance (Figure 5A). Among them, the curves for BMI and creatinine were closer to the upper-left corner, suggesting a higher discriminative ability in predicting the progression of T2DM patients to end-stage renal disease. The composite prediction model constructed from the multivariate logistic regression analysis demonstrated relatively superior discriminative ability, with an AUC of 0.880 (95% CI = 0.850–0.910, Figure 5B). Based on this, we further developed a nomogram model (Figure 5C), incorporating the predictors BMI, diastolic blood pressure (DBP), hematocrit (HCT), albumin (ALB), and creatinine (CREA). The nomogram was validated using a calibration curve (Figure 5D). The Hosmer–Lemeshow goodness-of-fit test yielded a result of *p* = 0.156, and the calibration curve showed a high degree of alignment with the ideal fit line, indicating good model calibration. Furthermore, various machine learning models were constructed and compared using a 7:3 split for the training and testing sets (Figure 5E). The results showed that the logistic regression model (AUC = 0.870) outperformed the Support Vector Machine (SVM, AUC = 0.840), XGBoost (AUC = 0.820), and Random Forest (RF, AUC = 0.860) in predictive performance, suggesting that the multivariate logistic regression model offers a better overall balance between discriminative power and interpretability.

**Figure 6.**
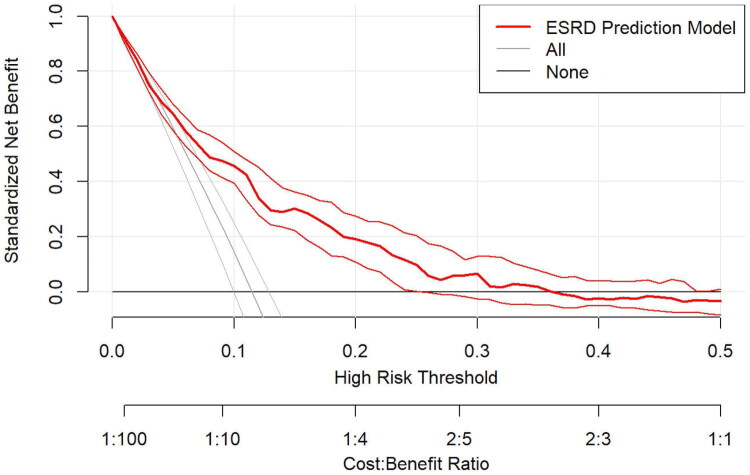
Decision curve analysis for the ESRD prediction model. The y‑axis represents standardized net benefit, and the x‑axis represents high‑risk threshold probability. The red line shows the net benefit of the prediction model, the gray dashed line represents the ‘treat‑all’ strategy, and the black line represents the ‘treat‑none’ strategy. The model demonstrates positive net benefit from 2% to 46% and exceeds both extreme strategies within the optimal range of 2%–26%.

The decision curve analysis demonstrated that the ESRD prediction model provided a positive net benefit across threshold probabilities ranging from approximately 2% to 26%, exceeding both the ‘treat-all’ and ‘treat-none’ strategies. The model showed optimal clinical utility at threshold probabilities between 10% and 20%, which corresponds to clinically relevant risk levels for ESRD prevention (Figure 6).

The logistic regression model demonstrated good discriminative ability with an AUC of 0.878. Bootstrap internal validation (500 resamples) confirmed the robustness of the model, yielding an optimism-corrected AUC of 0.878 (95% CI: 0.850-0.908), with a standard error of 0.015. The narrow confidence interval and the absence of overfitting (original AUC = bootstrap-corrected AUC) support the stability and generalizability of the model within the study population.

Further analysis revealed that the AUC values for all models were greater than 0.800, indicating relatively high predictive accuracy. Among them, the Logistic Regression model had the largest AUC value, demonstrating overall superior discriminative ability compared to the other models. Although the Random Forest and Support Vector Machine models performed well in terms of sensitivity, their specificities were 80.950% and 78.570% respectively, showing a certain gap compared to the Logistic Regression model (Table 6). In summary, the Logistic Regression model holds an advantage over the other models in terms of comprehensive predictive performance.

**Table 6.** Comparison of machine learning model performance.

	AUC	Sensitivity	Specificity
Logistic	0.870	76.210%	88.100%
XGBoost	0.820	56.560%	92.860%
Random Forest	0.860	80.090%	80.950%
SVM	0.840	80.540%	78.570%

## 4. Discussion

This study, integrating multivariable Mendelian Randomization and retrospective cohort analysis, investigated the key factors associated with the progression from T2DM to ESRD from both genetic and clinical perspectives. The results indicate that elevated BMI and creatinine levels significantly increase the risk of ESRD, while a higher hematocrit exhibits a protective effect. This association remained consistent in both multivariable MR analysis and multivariate logistic regression. The constructed multivariate prediction model demonstrated high discriminatory power and was validated through a nomogram and various machine learning models, showing good stability and interpretability. The integrated results suggest that elevated BMI is a major risk factor for the progression from T2DM to ESRD, while a higher hematocrit serves as a protective factor. These findings may provide a reference for early risk assessment and intervention in diabetic kidney disease.

The results of this study are largely consistent with previous research. Au et al. [27] reported that nonalcoholic fatty liver disease can influence T2DM progression, while Zheng et al. [28] revealed a causal relationship between cardiometabolic abnormalities and chronic kidney disease, both noting that racial differences might contribute to heterogeneity in MR results. To minimize the confounding effect of population structure, this study strictly controlled the sample source, selecting individuals of East Asian ancestry to reduce the impact of racial variation. The results showed that the associations of BMI and hematocrit with the progression from T2DM to ESRD remained significant in this population. Several studies support these findings: Chao et al. [29] confirmed that elevated BMI significantly increases ESRD risk; Doi et al. [30] found that canagliflozin significantly reduced ESRD risk by increasing hematocrit and decreasing the urine albumin-to-creatinine ratio (UACR). Notably, the consistent findings regarding BMI with ESRD risk suggest they may be universal risk factors across ethnicities. The mechanism of BMI is more complex, potentially influenced by metabolic status, insulin resistance, and body fat distribution [31,32], and requires further validation in larger, multi-ethnic cohorts.

Mechanistically, elevated BMI are closely linked to the progression from T2DM to ESRD, involving multiple pathways including metabolism, inflammation, and hemodynamics. Obesity promotes glomerular hypertension, hyperfiltration, and fibrosis through insulin resistance, chronic inflammation, and adipokine action. Concurrently, elevated BMI is positively correlated with creatinine levels, reflecting impaired renal function [33–35]. In contrast, a HCT appears protective, possibly reflecting enhanced erythropoietin (EPO) activity, improving tissue oxygen delivery and mitigating microvascular hypoxic injury [36,37]. Obesity is often associated with lower HCT; maintaining or moderately increasing HCT may partially counteract the renal risks associated with obesity and high creatinine [38]. Therefore, obesity, HCT form an interconnected mechanistic network, offering potential targets for intervening in T2DM progression.

From a clinical perspective, the findings of this study suggest that in the comprehensive management of patients with type 2 diabetes mellitus (T2DM), in addition to routine glycemic control, attention should also be focused on weight management, renal function monitoring, and changes in hematocrit levels. BMI and creatinine levels can serve as early warning signals of disease progression, indicating that patients require closer follow‑up and intervention. Research has shown that moderate caloric restriction (approximately 25%) can effectively delay the decline in early GFR in T2DM patients, thereby improving renal function [39]. In response to the early warning signal of elevated BMI, glucagon‑like peptide‑1 (GLP‑1) receptor agonists have recently become an important therapeutic option for weight management in T2DM patients with CKD, owing to their significant weight‑reducing effects and independent kidney‑protective actions. The landmark FLOW trial demonstrated that in T2DM patients with CKD, semaglutide reduced the risk of the primary composite kidney outcome by 24%, leading the FDA to approve it for reducing the risk of kidney failure [40–41]. The AWARD‑7 trial showed that dulaglutide reduced the risk of *a* ≥ 40% decline in estimated GFR (eGFR) or end‑stage kidney disease (ESKD) by approximately 55% compared with insulin, with a particularly pronounced benefit in the macroalbuminuria subgroup (hazard ratio 0.25) [42]. The REMODEL mechanistic trial further revealed that semaglutide reduces intrarenal and perirenal fat (by 25% and 13%, respectively) and downregulates glomerular endothelial cell metabolic stress gene expression by 63% and fibro‑inflammatory gene expression by 60%, thereby improving microcirculation and glomerular hemodynamics [43]. These lines of evidence indicate that lowering BMI with GLP‑1 receptor agonists not only directly targets the high‑BMI risk identified in this study but also confers additional kidney benefits independent of weight loss. Therefore, for T2DM patients with obesity or overweight, priority should be given to GLP‑1 receptor agonists to simultaneously achieve weight management and kidney protection. At the same time, the conclusion from this study that ‘maintaining an appropriate hematocrit level may have a protective effect’ must be interpreted cautiously against the backdrop of the cardiovascular safety concerns associated with erythropoiesis‑stimulating agents (ESAs). ESAs have long been associated with significant risks in CKD patients: the TREAT trial showed that darbepoetin alfa nearly doubled the risk of stroke in T2DM patients with CKD (5.0% vs. 2.6%) [44]. Multiple meta‑analyses have consistently confirmed that targeting hemoglobin levels above 11 g/dL significantly increases the risks of myocardial infarction, stroke, venous thromboembolism, heart failure hospitalization, and all‑cause mortality [45]. A key distinction is that the increase in hematocrit observed in this study results from endogenous, physiological EPO upregulation induced by SGLT2 inhibitors reducing proximal tubular metabolic load and oxidative stress, rather than from pharmacological stimulation with exogenous, high‑dose ESAs. This mechanistic difference may explain why SGLT2 inhibitors and GLP‑1 receptor agonists improve kidney outcomes without exhibiting the cardiovascular adverse events associated with ESAs. Consequently, in clinical practice, a strict distinction should be made between ‘drug‑induced physiological increases in hematocrit’ and ‘ESA‑driven obligatory increases’ – the former has safety and kidney‑protective potential within a certain range, whereas the latter warrants vigilance regarding cardiovascular risk.

This study has certain limitations. First, the sample size and diversity of East Asian populations in GWAS are still lagging behind European populations, which may limit the breadth and generalizability of the findings. Second, the cohort data contained a small amount of missing values; although imputation methods were applied, potential bias may remain. Third, this study did not encompass all potentially relevant biochemical indicators or longitudinal temporal data, so the inference of causal relationships may not be exhaustive. In addition, the study is primarily based on statistical methodologies and cannot elucidate the specific biological mechanisms underlying disease progression, necessitating further investigation through functional experiments in the future. Furthermore, the retrospective cohort did not employ time‑dependent models, which results in an insufficient description of the dynamic nature of disease progression. Finally, an important methodological consideration is the difference in outcome definitions between the MR analysis and the clinical cohort. Specifically, due to the availability of large‑scale GWAS data, chronic renal failure (CRF) was used as the outcome in the MR analysis, whereas ESRD was defined as the clinical endpoint in the retrospective cohort. Although CRF and ESRD are not identical clinical entities, they represent different stages of CKD progression: CRF reflects earlier or intermediate renal dysfunction, while ESRD represents the terminal stage requiring renal replacement therapy. From a pathophysiological perspective, the transition from CRF to ESRD involves a continuous decline in kidney function driven by shared mechanisms such as metabolic dysregulation, hemodynamic changes, and chronic inflammation. Thus, the MR analysis primarily captures genetic susceptibility to kidney function impairment, whereas the cohort analysis reflects clinical progression to advanced renal failure. Notably, the consistency of key risk factors—including body mass index and hematocrit—across the two analytical approaches supports the reliability of our findings across different stages of disease progression. However, the difference in outcome definitions may introduce some heterogeneity and should be considered a limitation. Future studies using GWAS datasets specifically for ESRD or longitudinal datasets covering the entire trajectory of CKD progression are necessary to further validate these findings. Overall, this integrated design provides complementary insights into the genetic susceptibility and clinical evolution of diabetic kidney disease, despite inherent differences in outcome definitions.

In summary, this study indicates that body mass index and hematocrit are significant factors associated with the progression from T2DM to ESRD, with findings from Mendelian randomization analyses being consistent with a potential causal effect and supported by clinical validation. These results suggest the potential utility of these indicators in risk stratification and clinical prediction. However, given the inherent limitations of MR and retrospective analyses, caution is warranted in interpreting causality. The proposed nomogram requires further validation in external, multi-center, and multi-ethnic cohorts. In addition, prospective studies are needed to evaluate whether interventions guided by this risk stratification model can effectively delay or prevent the progression to ESRD. Such efforts will help to strengthen the evidence base for precise prevention and individualized management of patients with T2DM.

## Data Availability

The data that support the findings of this study are available on request from the corresponding author, B. The data are not publicly available due to ethical and privacy concerns, as approved by the Ethics Committee of Qingpu Branch of Zhongshan Hospital Affiliated to Fudan University.
